# Atypical localized Mongolian spots in dark pigmented skin - a challenge for forensic medical examination

**DOI:** 10.1007/s00414-024-03248-6

**Published:** 2024-06-01

**Authors:** Stefanie Schlepper, M. Hagen, R. Schulz, A. Schmeling

**Affiliations:** Institute of Legal Medicine, University Hospital Münster, Münster, Germany

**Keywords:** Mongolian spots, Child abuse, Assessment of dark pigmented skin, Hematomas

## Abstract

The assessment of skin changes in the context of possible child abuse is an important task in forensic medicine. This requires knowledge of pigmentation variants and pigmentation disorders such as congenital dermal melanocytosis, which includes Mongolian spots. Particularly in the case of atypical localization and dark pigmented skin, the differentiation from hematomas can be challenging. A case of two Nigerian siblings with extensive and atypically localized Mongolian spots is reported. The 1.5-year-old girl showed Mongolian spots on her back and the right side of her trunk. The 8-year-old boy showed Mongolian spots only on the back of his thighs. The authors are not aware of any case in which so called Mongolian spots were present exclusively on the back of the thighs and this case is all the more noteworthy as the back of the thigh is a common localization of blunt force trauma.

## Introduction

Worldwide, 18% of children are subjected to physical violence every year [1]. The most common cause is blunt force trauma, which can result in typical skin findings such as hematomas, abrasions or lacerations. In the assessment of child abuse, a forensic medical examination to evaluate injuries serves as an important element. The responsible institutions (youth welfare office/police) often approach forensic experts to clarify what caused the injuries and whether those injuries are the result of physical child abuse. However, before child abuse can be suspected, it must be clarified whether a medical condition or an accident may have been the cause.

The assessment of dark pigmented skin presents a particular challenge. In addition to the lower visibility of skin hemorrhages, knowledge of various pigmentation disorders is of great importance in order to avoid misinterpretations. In addition to pigmentation variants such as pigmentary demarcation lines, local hyper- or hypopigmentation and the linea alba or linea negra, also pathological pigment disorders may be present. Primarily to be considered and clarified in this context are congenital dermal melanocytoses. These include Mongolian spots, the nevus of Ito and the nevus of Ota [2].

Mongolian spots are singular or multiple congenital skin discolorations that vary greatly in their morphology and coloration [3]. They are caused by the incomplete migration of melanocytes from the neural crest into the skin during the 11th − 14th week of pregnancy. The accumulation of melanocytes results in small to large, roundish, spindle-shaped or irregularly shaped skin discolorations. Mongolian spots are predominantly localized in the lumbosacral region (82.6%). In rare cases, the extremities (0.98%), buttocks (11.03%), shoulders (1.47%) or trunk (3.92%) are affected [4] - these are to be considered atypical Mongolian spots. Although a malignant degeneration risk is not known, Mongolian spots may represent dermal signs of various inherited disorders [5–9].

Nevus of Ito is a similar appearance that affects the shoulder and upper chest region and occurs primarily in Japan [10]. The nevus of Ota is predominantly localized on the face, unilaterally in the supply area of the 1st and 2nd branch of the trigeminal nerve and can show eye involvement with pigmentation of the sclera. It can occur both congenitally and during puberty or pregnancy, suggesting an association with hormonal changes [10].

This case report is about an 8-year-old boy and his 1.5-year-old sister who were presented to forensic medicine regarding suspected physical child abuse.

## Case report

The youth welfare office initially contacted the institute of forensic medicine with a request to examine an 8-year-old Nigerian boy. He has been taken into custody three days prior and was placed in a residential group. However, the staff at the residential group had noticed bruises on the back of both thighs while he was showering.

**Fig. 1:**
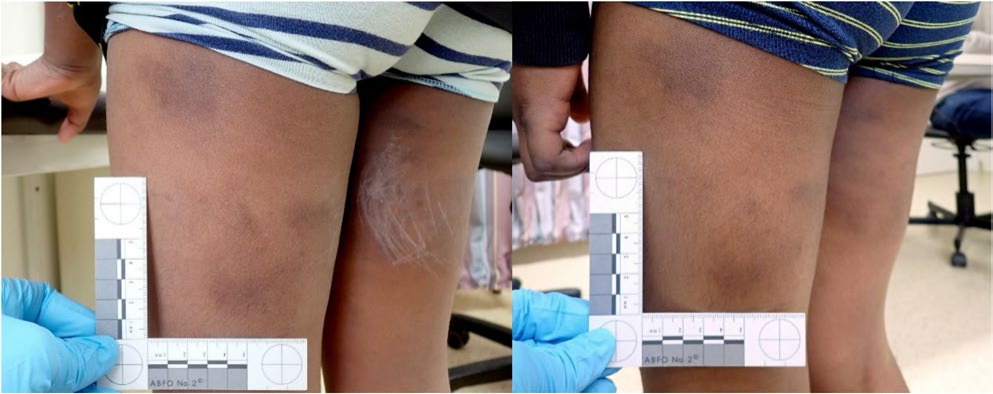
8-year-old boy with dark macules on the back of his left thigh during the first forensic medical examination and 1.5 months later

Upon forensic medical examination, the 8-year-old, dark-skinned (skin type 6 of the Fitzpatrick classification [11]) boy was found to be in good nutritional, nursing and general condition. Two dark blue to purple skin discolorations up to 7 cm in size were found on the back of the left thigh (Fig. 1). On the back of the right thigh, there were several small to large, dark blue to purple skin discolorations on an area measuring 10 × 8 cm. Child abuse by blunt force was initially suspected.

After 2 weeks, the youth welfare office contacted the institute of forensic medicine again requesting a forensic examination of the boy’s 1.5-year-old Nigerian sister. She had previously been in the care of the child’s mother and during a visit by the Youth Welfare Office, abnormalities were found on the back of her torso.

**Fig. 2:**
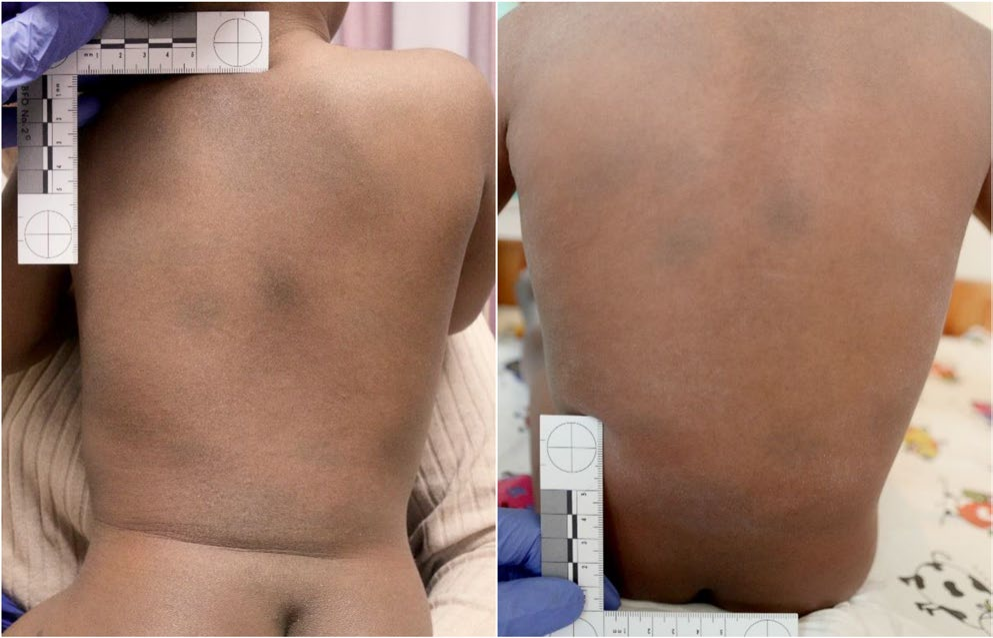
1.5-year-old girl with dark macules on his back during the first forensic medical examination and 2 weeks later

Upon forensic examination, the 1.5-year-old, dark-skinned (skin type 6 of the Fitzpatrick classification [11]) girl was found to be in good nutritional, nursing and general condition. The back of the trunk showed a total of four dark skin discolorations, up to 4 cm in size, in the area of the right, rear shoulder region, the middle and lower back region and the right side of the trunk (Fig. 2). The skin of the lower back was discolored bluish across a large area. The skin discolorations were classified suspicious with regard to physical maltreatment, however a possible pigment disorder such as Mongolian spots was discussed as a differential diagnosis. Therefore, a second forensic medical examination was carried out two weeks later. The skin discoloration remained unchanged and the examined birth report documented “pigment spots on the back”. Consequently, the skin findings could be classified as Mongolian spots.

Based on this, a second examination of the 8-year-old boy was carried out one and a half months after the initial examination and his skin discolorations also remained unchanged.

Ultimately, there was no evidence of child abuse through blunt force trauma in either the 8-year-old boy or the 1.5-year-old sister.

## Discussion

The assessment of non-white skin can pose a challenge for the examiner. In addition to a lower visibility of skin hemorrhages and the associated urgency of an examination, pigmentation disorders such as congenital dermal melanocytosis must be considered as a differential diagnosis [10].

The prevalence of Mongolian spots is subject to a wide range. While they occur in > 90% of US-African American and Asian newborns, less than 10% of Europeans are affected [12]. The siblings presented here are from Nigeria, where the prevalence of Mongolian spot has been described as 44.7% (74.8% in newborns, 13.6% in preschoolers) [13].

Mongolian spots are easily recognized if they are typically located in the lumbosacral region. However, atypical localizations must also be considered and reflected upon assessment. Kettner et al. were able to detect Mongolian spots at different locations in 10.27% of 253 children examined. The authors developed a classification scheme according to localization (I - buttocks, II - not buttocks, III - combination of I and II) with several subtypes [14].

The 1.5-year-old girl presented Mongolian spots on the back and on the right side of the trunk, which according to the classification of Kettner et al. [14] can be categorized as type III. Kolbe et al. [15] reported a 2-month-old girl with extensive Mongolian spots on the back of the trunk, buttocks and both ankles. Diers et al. [16] also reported a 2-month-old girl with Mongolian spots on the buttocks, right shoulder, both lower legs and the back of the foot. Further case reports on Mongolian spots with atypical localization have been published [17–19].

An association of extensive Mongolian spots in atypical localizations with inherited disorders such as GM1 gangliosidosis, Hurler’s syndrome or Hunter’s syndrome has been described [5, 7]. In the present case, however, such a disease was not present.

The Mongolian spots of the older brother represent a very rare localization. The authors are not aware of any case in which Mongolian spots were present exclusively on the back of the thighs and this case is all the more noteworthy as the back of the thigh is a common localization of blunt force trauma.

The boys age (8 years at the time of the study) is also unusual. While Mongolian spots predominantly disappear during the first years of life, persistence into adulthood is extremely rare [3, 19]. Onayemi et al. [13] examined a total of 853 children and newborns in Nigeria. No child older than 6 years had Mongolian spots.

Furthermore, it is remarkable that there were two siblings with atypical Mongolian spots. The extent to which Mongolian spots run in families is not known with certainty. Beeregowda et al. [20] presented an Indian family, 13 of whose members had pronounced Mongolian spots and concluded an autosomal dominant inheritance.

Differentiating (pathological) pigmentation variants from hematomas can easily be done by means of a follow-up examination. While “real” hematomas are subject to physiological degradation processes, pigmentation variants persist for months - years. If a possible congenital pigmentation disorder is suspected, a follow-up forensic medical examination should be carried out a few weeks after the initial examination; Kettner et al. [14] recommend an interval of 2 to 6 weeks between the two examinations. For optimal comparability and thus a well-founded assessment, photo documentation of the skin abnormalities should be carried out under the same conditions.

Recognizing child abuse is of great importance, but also the knowledge of differential diagnoses such as congenital dermal melanocytosis to prevent the misdiagnosis of child abuse.

## Data Availability

The authors confirm that the data supporting the findings of this study are available within the article.
